# Translating Material Science into Bone Regenerative Medicine Applications: State-of-The Art Methods and Protocols

**DOI:** 10.3390/ijms23169493

**Published:** 2022-08-22

**Authors:** Lorena Di Pietro, Valentina Palmieri, Massimiliano Papi, Wanda Lattanzi

**Affiliations:** 1 Dipartimento Scienze della Vita e Sanità Pubblica, Università Cattolica del Sacro Cuore, Largo F. Vito 1, 00168 Rome, Italy; 2 Fondazione Policlinico Universitario “A. Gemelli”, IRCSS, Largo A. Gemelli, 00168 Rome, Italy; 3 Institute for Complex Systems, National Research Council of Italy (CNR), Via dei Taurini 19, 00185 Rome, Italy; 4 Dipartimento di Neuroscienze, Università Cattolica del Sacro Cuore, Largo F. Vito 1, 00168 Rome, Italy

**Keywords:** bone scaffold, tissue regeneration, protocols, methods

## Abstract

In the last 20 years, bone regenerative research has experienced exponential growth thanks to the discovery of new nanomaterials and improved manufacturing technologies that have emerged in the biomedical field. This revolution demands standardization of methods employed for biomaterials characterization in order to achieve comparable, interoperable, and reproducible results. The exploited methods for characterization span from biophysics and biochemical techniques, including microscopy and spectroscopy, functional assays for biological properties, and molecular profiling. This review aims to provide scholars with a rapid handbook collecting multidisciplinary methods for bone substitute R&D and validation, getting sources from an up-to-date and comprehensive examination of the scientific landscape.

## 1. Introduction

Bone is the second most commonly transplanted tissue after blood, and bone reconstructive and regenerative strategies have been the focus of an ever-growing area of scientific research in the field.

The adult human skeleton is composed of around 206 bones and is in charge of diversified physiological organism functions: locomotion and mechanical, shape of the body cartilage, connective and adipose tissues, plus vessels and nerves. Bone fractures represent a significant global health challenge, being the most common traumatic injuries affecting large organs in humans, with variable incidence and prevalence rates in different regions, ages, and nosological categories [1]. Despite the bone’s ability to regenerate and repair itself, approximately 5–10% of patients suffering from bone fractures may experience healing failure, leading to functional impairment, chronic pain, and disability, with great negative impact on the overall quality of life and on the related health economic burden [2].

According to the classical triangular biological model, successful bone healing requires the co-presence of three essential components: osteoinduction, osteogenesis, and osteoconduction. Osteoinduction is the role played by bioactive signals able to induce osteogenic cells to form new bone [3]; osteogenesis is the process through which stem cells commit, proliferate, and differentiate towards an osteoblastic lineage and start producing extracellular matrix (ECM); osteoconduction is the growth of bone on the appropriate surface within a tridimensional architecture [3,4]. Besides this triad, the role of mechanical load and the environment surrounding the healing bone is a fourth parameter that can be considered, defining the so-called diamond-shaped concept in bone healing [5].

Whenever a bone fails to regenerate on its own, a bone grafting strategy must be adopted, that is, an implanted tissue/material that promotes bone healing. The selection of the ideal bone graft depends on different intrinsic and extrinsic factors, including the patient’s age and co-morbidities, tissue viability, defect size and anatomical location, along with graft chemical composition, biomechanical features, handling, and cost. The materials used in bone grafting can be divided into three gross categories: autografts, allografts, and xenografts, each including several types and showing advantages and disadvantages. Despite representing the ‘gold standard’ of autografts, the pain and donor site morbidity associated with their harvesting represent the main disadvantages of their use [4]. For these reasons, several alternative synthetic and biologically based tissue-engineered biomaterials have been introduced into clinical practice, and an increasing variety of biomaterial compositions are being proposed as alternative strategies on a daily basis by scientific research in the field [6].

With the implementation of 3D culture systems, biomaterial synthesis, and 3D printing, the characterization and design of regenerative medicine scaffolds demands a multidisciplinary approach ranging from biophysical and biomechanical studies to applied biology. This concise primer intends to offer an overview of methods currently available for bone regenerative scaffolds characterization.

We will first discuss the importance of bulk material composition, morphology, and mechanical properties; then we will move to discuss the surface role in the adsorption of relevant macromolecules after implantation in vivo. Then, finally, we will systematically detail the biological assays aimed at testing and validating the suitability and biologically functional effectiveness of a bone substitute material. 

## 2. Bone Scaffold Architecture: Characterizing Scaffold Internal Structure and Composition through Spectroscopy, Microscopy, and Mechanical Testing

Materials used for bone scaffolds engineering include natural and synthetic polymers, bioceramics and bioglasses, and composite materials [7,8,9]. In Table 1, the advantages and limitations of different types of grafts are summarized [10]. Bone grafts can be obtained from autologous bone, which is still the gold standard for the lack of immunogenicity despite the additional surgery required. Xenografts are implants derived from other species, such as bovines, while xenohybrids are a combination of synthetic materials with bovine, porcine, or equine xenografts. The addition of nanomaterials, especially carbon-based nanoparticles such as carbon nanotubes and graphene, has been exploited to: (i) control cell attachment [11], (ii) modulate mechanical properties [12,13], (iii) control scaffold bioactivity by external stimuli such as infrared light, and (iv) develop coatings that limit scaffold brittleness and local inflammation [14].

Evaluating the effects of composition on microstructural and osteoconductive properties of biomaterials is fundamental. Material chemistry influences hydrophilicity, charge, and interaction with biomacromolecules in patients. Interestingly, it has been demonstrated that osteogenic differentiation of human mesenchymal stromal cells (hMSCs) is greatly improved when chemical factors are combined with physical cues, i.e., the surface microtopography, in order to induce mechanosensing and hence mechanotransduction response in the cells [15]. Chemical characterization is performed by techniques such as Fourier transform infrared spectroscopy, Raman and X-ray photoelectron spectroscopy, ultraviolet visible spectroscopy, nuclear magnetic resonance, and mass spectrometry [16]. According to the type of material, infrared spectroscopy can identify inorganic and organic structures, providing information on the functional groups. Raman spectroscopy can be used to supplement the IR data and can be especially useful in aqueous solutions. X-ray diffraction (XRD) analysis can be used to establish the type of ceramic or metallic material and phase analysis of ceramic materials.

Besides specific material composition, the above-mentioned techniques allow for the evaluation of bone matrix formation when cells are grown on the scaffold. Indeed, bone matrix characterization involves quantification of minerals like carbonated hydroxyapatite, collagen (mainly type I), non-collagenous proteins, and proteoglycans [17]. For a review of the different techniques used to characterize the chemical and structural properties of materials, see [18].

Chemical properties and biological interactions influence scaffolds resorption that may result in vivo from the activity of macrophages and osteoclasts. Indeed, (biodegradable) scaffolds should gradually degrade with time with a resorption rate that matches new bone formation until wholly replaced by new tissue [19]. It is commonly thought that a scaffold should maintain strength for at least 4 months (the range varies according to the implant site and the age and morbidities of the recipient patient), given that the healing of bone tissue includes an early inflammatory stage (3–7 days), the repair stage (3–4 months), and finally the continuous remodeling stage (months to years) [20]. The degradation rate is usually quantified in simulated body fluid by measuring weight loss [21]. Phenomena occurring during scaffold resorption are also discussed in the next chapter.

From a structural point of view, the hierarchical bone architecture constantly undergoes renewal and repair [17]. Reznikov and colleagues divided lamellar bone into nine structural levels from the organ to the components collagen, hydroxyapatite, glycosaminoglycans, and non-collagenous proteins [17]. 

Scaffold replicating bone architecture either recapitulates structural features or induces the correct replacement of implanted material in vivo according to the natural hierarchical structure. New fabrication techniques such as 3D printing promise to fulfill bone architectural demands, at least at the macroscale (i.e., micrometric resolution) [4,22,23]. Recent reviews on 3D printing for bone reconstruction delve deeper into the topic [4,24]. The physical characterization of newly formed bone structure is principally obtained by histological examination after in vivo implantation while microscopy such as scanning electron microscopy (SEM) is typically used to evaluate scaffolds prior to implantation. SEM is indeed one of the most important and diffused microscopy techniques and is based on the scanning of the surface of materials with an electronic beam, reaching a nanometric resolution. SEM is fast compared to other scanning techniques such as atomic force microscopy, though the sample preparation can be destructive to cope with the vacuum environment in the microscope chamber. Recent SEM instruments are equipped with scanning transmission electron microscopy (STEM) detectors, which considerably extend their capabilities. A technical overview of SEM applied to nanostructured materials can be found in [25]. SEM can also be coupled with energy-dispersive X-ray spectroscopy (EDX), a technique that exploits the X-ray spectrum emitted by the sample to obtain site-specific chemical analysis with minimal sample preparation. In bone, the most frequent application of EDX is the measurement of extracellular matrix Ca and P content [26]. Interestingly, selective removal of specific bone components is a valuable approach for understanding an individual’s contribution to the overall morphology (for protocols see [26]).

SEM sample preparation is strictly dependent on material intrinsic properties since soft materials might collapse under vacuum without proper sample fixation.

Porosity, i.e., the percentage of void space in a solid, is an important feature of a scaffold to permit cell migration and proliferation, and vascularization. Porosity is around 5–10% in cortical bone and 75–90% in cancellous bone [27]. By applying pressure [16], porosity can be calculated using mercury intrusion porosimetry, a technique based on the ink injection of a nonwetting liquid through the pores of a scaffold [16]. Micro-computed tomography (micro-CT), a 3D X-ray-based technique, also allows quantification of porosity, pore size, and interconnectivity. Furthermore, Palmroth and colleagues exploited micro-CT to analyze the distribution of labelled human adipose-derived stem cells in scaffolds using different seeding methods [28].

Porous materials may have open and closed pores; the latter are not reachable by fluid. Conversely, open pores can be dead-end pores or interconnected/through pores, which make the passage of fluids feasible. Based on the pore diameter, scaffolds can have micropores (<2 nm), mesopores (2–50 nm), and macropores (>50 nm). Interconnected macropores are a crucial factor for bone regeneration, as they provide the environment for tissue in-growth [16]. Porogen leaching, gas foaming, freeze-drying, solution electrospinning, melt electrowriting, and 3D printing are all methods for creating pores in scaffolds, as illustrated in Figure 1 from [29]. 

The role of porosity in scaffold permeability to cells and vascularization has been comprehensively reviewed in [29].

Pores also influence the mechanical properties of scaffolds together with the biomaterial composition and deposition technique. The important mechanical properties of the bone include Young’s modulus, toughness, shear modulus, tensile strength, fatigue strength, and compressive strength [30]. Mechanical properties of bone vary between compact and spongy bone: indeed compact bone can withstand much higher stress (up to ~150 MPa) but lower strain (3%) before failure, while spongy bone can withstand lower stress (up to ~50 MPa) but much higher strain (50%) [31]. Mechanical testing of scaffolds involves uniaxial tension, compression, indentation, and dynamic mechanical [32]. Parameters obtained by the uniaxial tensile test are reported in Table 2 [31]. Note that these properties vary according to age and health condition, as is visible for comparison between normal and osteoporosis bone in Table 2. This should be considered for matching natural bone and graft properties, especially in elderly patients.

Material hardness can influence the choice of mechanical tests. Indeed, while some bone substitutes are designed for prolonged in vivo resistance, other materials such as hydrogels or fast resorbable composites are easily degraded and soft. For these types of samples, micropipette aspiration, atomic force microscopy (AFM)-based nanoindentation, and squeezing in microchannel confinement can be performed for characterization of mechanical properties [33].

AFM can indeed be used to quantify the micromechanical properties of biological materials that, at a cellular scale, are involved in guiding cell fate [34]. AFM is based on a scanning nanometric tip connected to a cantilever whose deflection is measured by an incident laser. AFM can image topography at high resolution and can be used to obtain force-distance curves on the surface. From these curves, parameters such as elasticity, hardness, adhesion, and surface charge can be quantified [35].

## 3. Biomaterial-Tissue Interface

**Figure 2:**
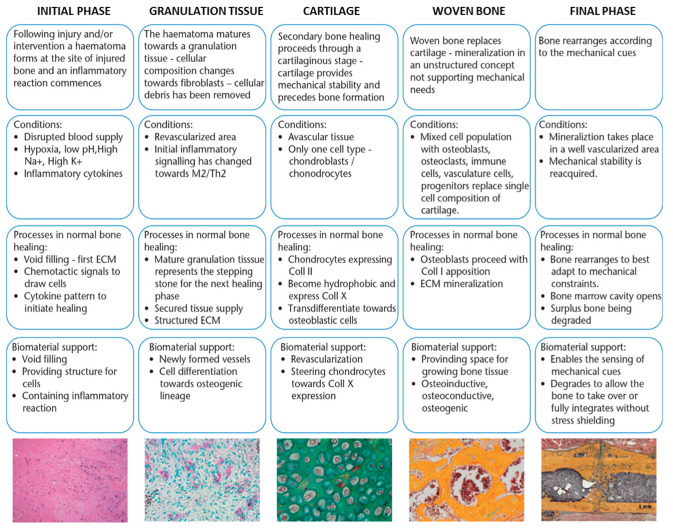
Phases of bone growth on biomaterial and necessary support features. Modified from [37] Creative Commons CC-BY license.

The surface properties of biomaterials represent a key feature in driving cell-scaffold interactions and, as a result, mediating appropriate biological response after implant. In some types of implants, the surface can be inert, i.e., neither protein nor cells can attach, so that blood coagulation and thrombosis are impeded. This approach is feasible for heart valves, vascular prostheses, catheters, and hemodialysis tubes but would impede the osteointegration of bone grafts. Conversely, bone scaffolds are usually required to promote cell adhesion in order for cells to proliferate and differentiate [36]. The wound healing process in bone regeneration requires an initial hematoma and inflammatory phase (Figure 2). Subsequently, the granular tissue transforms into a soft callus of cartilage that is mineralized to form the hard callus. Finally, the woven bone develops and remodels. 

Biomaterial properties should accompany all these phases, initially by sustaining cell growth and inflammation, then by supporting vascularization and differentiation, and finally by degrading to leave space for bone formation. 

**Figure 3:**
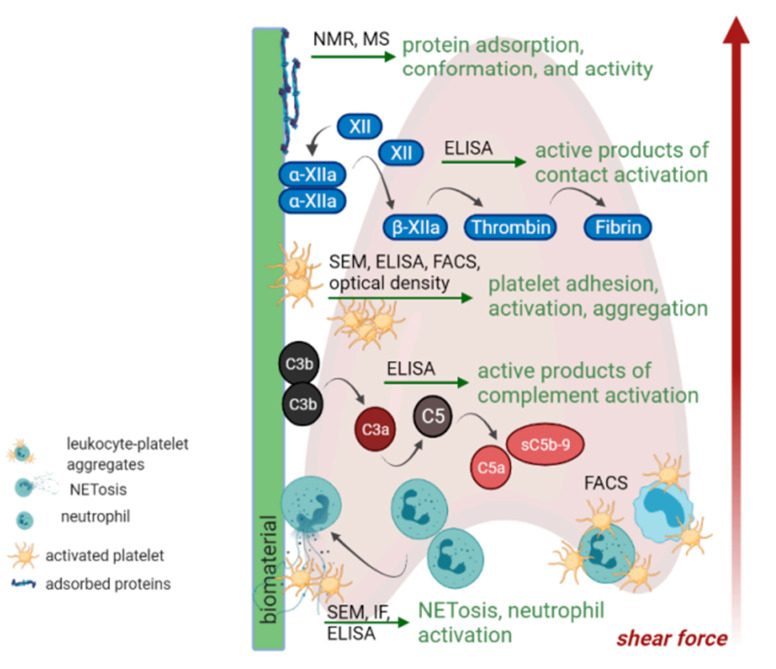
In vitro tests for blood compatibility. Reproduced from [38] Creative Commons CC-BY license. Abbreviations: Nuclear Magnetic Resonance (NMR), Mass Spectrometry (MS), Enzyme-linked immunosorbent assay (ELISA), Fluorescence activated cell sorting (FACS), Scanning Electron Microscopy (SEM), Immunofluorescence (IF).

For predicting the in vivo performance of a biomaterial in contact with blood, hemolysis, coagulation, platelet, complement, and leukocyte activation are fundamental for blood compatibility testing. Figure 3 gives an overview of possible targets of blood compatibility testing. However, standardized protocols or standard operation procedures and the specification of cell models are still lacking [38]. 

Materials’ surface features influence the interaction with cells by varying charge, hydrophobicity, and protein adsorption in vivo. Material wettability and hydrophilicity are generally measured by contact angle analysis. The contact angle is defined between the surface and the tangent line at the contact point of a water drop deposited on the material and represents a quantitative measurement of hydrophilicity [16]. 

**Figure 4:**
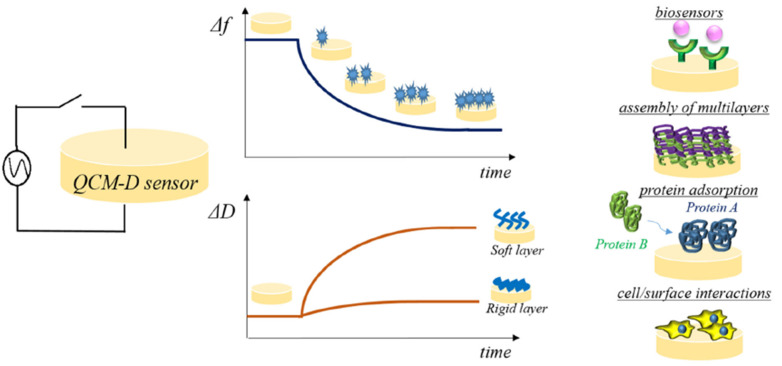
Experimental settings on QCM-D. Reproduced from [43] Creative Commons CC-BY license.

Immediately after implantation in the body, a conditional layer of proteins adsorbs on the surface, and this will influence eukaryotic cells’ (and bacteria’s) responses to the biomaterial [39]. Methods for studying protein adsorption have been recently reviewed in a practical review [40]. Driving forces for surface adsorption include electrostatic, hydrophobic, and van der Waals interactions and hydrogen bonds. In addition, the total amount of protein, and other parameters used to describe the type of adsorption are the conformational and aggregation state and the reversibility of the phenomenon. The enzyme-linked immunosorbent assay (ELISA), sometimes referred to as EIA (Enzyme Immuno Assay), is the most widely used test in biology to study protein concentration. Proteins are recognized by a specific antigen-antibody reaction and quantified by enzyme activity. Other widely used techniques for protein quantification are the Bicinchoninic acid assay and the Bradford assay, which are in turn not specific and can only describe the total protein amount adsorbed on the sample. For these assays, it is crucial to optimize the protocol to recover protein attached to the biomaterial by choosing the appropriate desorption buffer, as tested by Kratz and colleagues [41]. AFM can be used to image proteins attached to the surface or other nano- and micro-topographical features of the biomaterial at high resolution. Optical-based techniques used for protein adsorption quantification include surface plasmon resonance, optical waveguide lightmode spectroscopy, dual polarization interferometry, fourier transform infrared spectroscopy in attenuated total reflection mode, and spectroscopic ellipsometry [40]. The quartz crystal balance with dissipation monitoring (QCM-D) took hold in the field for the ease of measurement of protein adsorption kinetics. Real-time measurement of phenomena occurring at the interface between surfaces and biological fluids can be measured, including, protein adsorption [42], cell attachment under flow, platelet activation and biomaterial degradation over time (Figure 4) [43].

In vitro models of protein adsorption generally focus on principal plasma components: fibrinogen, whose concentration and unfolding is directly proportional to platelet activation, together with fibronectin, and vitronectin [38]. Circular dichroism (CD) spectroscopy is the gold standard for the evaluation of protein conformational changes on the surface. Studies with differentiated monocyte THP-1 cells demonstrated that immune cells interacted with surface adsorbed albumin, which could bind to exposed peptide sequences caused by surface induced unfolding (evaluated by CD). On the other hand, surfaces pre-treated with albumin induce the production of anti-inflammatory markers by immune cells [44]. Similarly, heparin coatings inhibit inflammation [45]. It is noteworthy to mention that in vitro systems simplify the complex formation of protein layers that occur in plasma. A dedicated field of study is focused on the so-called biomolecular corona of nanosystems and the conditional layer (CL) on the scaffold surface [46]. The balance of protein composition in the CL goes along with modulations of plasma protein levels. Various diseases, as well as immunosenescence and lifestyle factors, can cause variations in the CL composition of the plasma proteome and/or the conformation of proteins. Dysfunctions of the immune system, such as age- or disease-related changes in extracellular matrix, variation of cell accessories or of molecules concentration can affect tissue regeneration in implant sites and have been observed in patients [47,48].

Finally, it should be pointed out that the intrinsic properties of biomaterials can affect the selection of characterization methods and techniques. The performance of a material surface is often related to a critical material property, such as surface topography or purity. Specific methods for studying protein adsorption on bioglasses, which have a faster modification, have been recently reviewed [49]. 

## 4. Biological Characterization of Bone Substitute Materials

After biomaterial implantation, there are several factors that have a great impact on the bone tissue repair, inter alia macrophage–osteoblast cross-talk, environmental soluble factors, and surface properties of the implant. Cells in bone tissues (i.e., osteoclast, osteoblast, osteocytes, and MSCs) are mechanosensitive and respond to biophysical factors in the environment. Differentiation of hMSCs on the surface of biomaterials may be influenced by the properties of the specific biomaterial, such as its elasticity and surface topography.

The ideal biomaterial for bone regeneration should not only be biocompatible and osteoconductive but also osteoinductive. They should be able to leverage the self-healing capabilities of the bone by: (i) providing the main structural, compositional, and biochemical cues for the formation of new tissue; (ii) engaging the host’s resident immune cells in the regenerative response; (iii) promoting the recruitment, proliferation, and differentiation of progenitor cells; and (iv) recovering an adequate local blood supply to support healing and remodeling.

**Figure 5:**
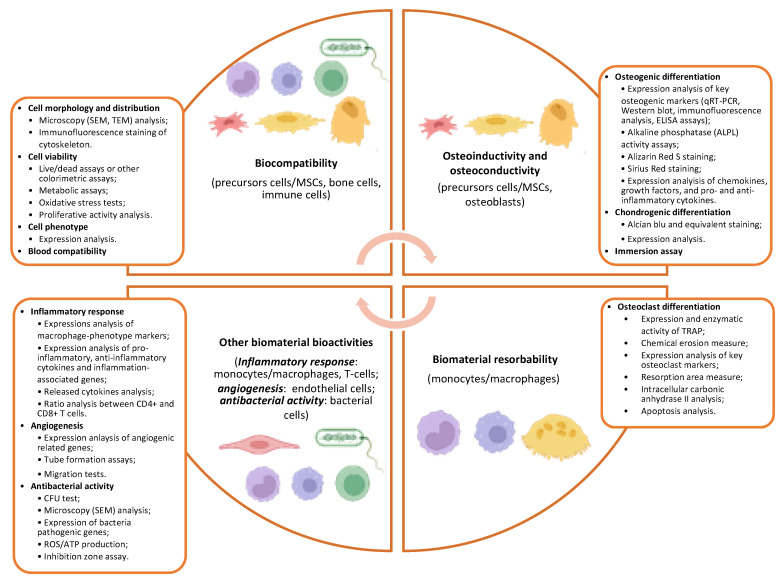
The scheme reports the most used methods to assess different biomaterial bioactive properties including biocompatibility, osteoinductivity, osteoconductivity, and resorbability.

Due to the large number and heterogeneity of the research articles on the topic and to the rapid obsolescence in applied biomaterial research, we have narrowed our analysis to the more recent studies published in the last 3 years to depict an up-to-date review of the topic. Figure 5 recapitulates the different methods described below to assess biomaterial effects on cell viability and differentiation.

## 5. Biomaterial Biocompatibility

The ideal materials for biomedical applications should meet the primary need of biocompatibility, which is defined as the capacity of the biomaterial to integrate within the local environment, ensuring cell viability and growth without eliciting any local and systemic detrimental responses such as immune, allergic, inflammatory and carcinogenic responses [50,51]. This involves a fine and complex interaction between the biomaterial and the biological environment at the implantation site that includes contact with the different cell types within the bone stem cell niche, including immune cells and blood cells, but also the adsorption of proteins and other secreted factors that inhabit the tissue.

There are several widely used methods to assess if cell viability is maintained in contact with the biomaterial and that cells can adhere and proliferate in culture. The choice depends on many factors, but it is certainly necessary to consider the transparency of the tested biomaterial because not all the analyses are suitable for a clear visualization of the cells on the scaffold. Cell localization and distribution on the biomaterial can be analyzed by simply labeling the nuclei at different timepoints after cell seeding on the scaffold [52]. The morphology of the biomaterial-seeded cells can also be examined by scanning electron microscopy (SEM) [14,53,54,55,56,57,58,59,60,61,62,63,64,65,66,67], as well as the intracellular structures by transmission electron microscopy (TEM) [65,68]. Cell morphology and distribution can also be assessed using immunofluorescence staining of actin filaments, by means of anti-phalloidin antibodies among others, which allows for the analysis of the cytoskeleton conformation of cell growth in contact with the biomaterials [14,52,54,55,57,60,62,64,65,69,70,71,72,73,74,75,76].

A qualitative and quantitative measure of cell viability can be assessed with different alternative methods. Fluorescent live/dead assays are able to discriminate between live and dead cells by evaluating plasma membrane integrity and the activity of the esterase enzyme, both maintained only in viable cells [54,55,60,62,70,74,75,77,78,79,80,81,82,83,84]. Other commercial colorimetric assays for the evaluation of cell viability are available on the market and, among the most widely used are the cell counting kit-8 (CCK-8) assay that allows a sensitive colorimetric measure of viable cells, using a water-soluble tetrazolium salt that produces, in presence of active dehydrogenases in living cells, an orange formazan product, and the amount of formazan produced is directly proportional to the number of viable cells [55,57,58,62,73,76,80,81,85,86,87,88,89]. Moreover, the Alamar Blue assay method is frequently used to assess the metabolic activity of proliferating cells: the active resazurin compound, upon entering living cells, is reduced to resorufin, a red fluorescent molecule that can be quantified [14,59,64,72,75,90,91]. 

The relative number of metabolically active cells seeded on the tested biomaterial is usually evaluated by the 3-(4, 5-dimethylthiazol-2-yl)-2, 5-diphenyltetrazolium bromide (MTT) colorimetric assay, based on the reduction of the yellow tetrazolium salt MTT to purple formazan crystals by NAD(P)H-dependent oxidoreductase enzymes present in metabolically active cells [14,56,60,61,65,68,82,92,93,94,95,96,97,98,99,100]. The enzyme lactate dehydrogenase (LDH) cytotoxicity assay is another widely used colorimetric assay that allows one to quantify the extracellular concentration of the LDH secreted upon cell damage [55,83,92,98]. The determination of the mitochondrial membrane potential represents another crucial parameter to assess the cell metabolism of scaffold-seeded cells [52,96]. The production of reactive oxygen species (ROS) can also be used to test for oxidative stress [59,67,101,102,103].

The proliferative activity of cells cultured with the biomaterial can be determined based on the distribution of cells in the cell cycle and DNA synthesis. The colorimetric assay based on the bromodeoxyuridine/5-bromo-2′-deoxyuridine (BrdU) or 5-ethynyl-2’-deoxyuridine (EdU) incorporation during the S-phase of the cell cycle of growing cells is amply used [52,57,70]. The cell cycle can also be analyzed using commercial kits able to measure G0/G1, S, and G2/M phase distributions considering the different staining of cells with propidium iodide (PI) by flow cytometry [52]. The advent of sophisticated live cell imaging equipment and software allows us to monitor cell adhesion, proliferation, and the capacity to spread out from the biomaterial after having attached to its surface by performing consequential imaging of viable cells in culture [65].

Other parameters can also be considered in order to study the effects of biomaterials on cell biology. To evaluate if the biomaterial also maintains the immunophenotype of the seeded cells, the expression of specific cell markers (for example, CD73, CD90, and CD105 for mesenchymal stromal cells) can be assessed by flow cytometry [104]. 

The expression of adhesion-related genes, namely *integrin subunit alpha 5* (*ITGA5*), *integrin subunit beta 3* (*ITGB3*), *integrin subunit alpha V* (*ITGAV*), *integrin subunit beta 1* (*ITGB1*), *protein tyrosine kinase 2* (*PTK2/FAK*), and *vinculin* (*VCL*), can be analyzed after culturing cells on biomaterials [74,105]. The use of focal adhesion staining kits is also reported [106].

## 6. Biomaterial Osteoinductivity and Osteoconductivity

### 6.1. Types of Cells

Different types of cells are tested in vitro to assess biomaterial biocompatibility and bioactivity for bone regenerative applications. MSCs are the ideal bone precursor cells for this aim. MSCs can be isolated from different tissues of both humans and animal models. They can proliferate and differentiate into osteoblasts upon appropriate stimuli. MSCs isolated from bone marrow are regarded as the gold standard [14,53,55,57,67,68,73,76,77,79,83,91,94,98,107,108,109]. MSC-like cells isolated from the stromal vascular fraction of the adipose tissue, namely, adipose-derived stem cells, ASC, are seldom used in selected applications [2,65,72,82,89,104,109,110,111,112]. MSCs isolated from the dental pulp also find a wide range of applications, but the application of this cell type is beyond the scope of this review. To overcome the limit due to the short number of passages for which these cells can be cultured, immortalized human bone marrow derived MSCs have also been used [113]. Other types of primary cells are also used in various studies such as primary osteoblasts obtained from long-bones [93]. 

Different cell lines also find an approved use in the field: the most used is the mouse calvarial preosteoblast MC3T3-E1 cell line [52,54,62,63,71,78,80,82,85,86,87,90,114]. Other reported cell lines are: human osteosarcoma SaOS-2 cells [56,64,88], the mouse osteoblastic KUSA-A1 cell line [84], the human osteoblast NHOst-Osteoblasts OGM cell line [66], and the human osteosarcoma MG63 osteoblast-like cells [58,95,97].

### 6.2. Osteogenesis

Several methods are used to evaluate the in vitro osteogenic differentiation capability of cells in contact with the scaffold. The osteogenic differentiative potential of cells is usually tested at different timepoints (from a few days up to 3 weeks) and compared with cells grown on cell culture vessels as controls. Some experiments are set by replacing the proliferative medium with an osteogenic medium or in others the intrinsic capacity of biomaterial to promote osteogenesis is directly assessed. Moreover, in this case, it is necessary to consider the transparency of the analyzed biomaterial to choose the most appropriate analysis to perform.

In the vast majority of papers in the literature, the expression levels of one or more genes associated with osteoblast differentiation are usually evaluated by quantitative real-time PCR (qRT-PCR) and/or Western blot and/or immunofluorescence analysis and/or ELISA assay [14,55,57,58,60,62,64,65,68,69,70,72,73,76,77,79,80,82,83,84,86,87,89,90,91,94,96,98,100,108,109,113]. Table 3 reports the most representative osteogenic genes usually analyzed. To better investigate the effects of biomaterials on cell metabolism and differentiation, it is also possible, using a RT-PCR array technology, to investigate the expression of numerous genes at once, coding for proteins involved in the osteogenic differentiation pathway, including bone mineralization, extracellular matrix modeling, or cell adhesion [72]. 

The analysis of alkaline phosphatase (ALPL) activity is one of the most common tests used to evaluate the osteogenic process and numerous easy-to-use and highly sensitive assays to measure ALPL activity are available on the market [14,53,54,55,58,61,65,66,67,68,70,71,73,76,79,80,83,85,86,88,90,91,93,94,95,96,97,98,99,107,114,115].

During osteogenic differentiation, calcium deposition can be measured by Alizarin Red S staining [14,53,54,55,57,61,64,65,67,68,69,71,72,76,83,84,85,86,87,90,91,93,94,96,99,114]. The direct visualization and analysis of the Alizarin Red stained deposits provides a qualitative measure of the mineralized matrix deposition, but it is possible only on transparent supports and not on thick opaque materials like ceramics and graphene composites. However, it is possible to quantify the amount of staining and compare different conditions by dissolving the Alizarin Red from the stained monolayer and obtaining a measure of the absorbance of each sample [14,53,57,61,64,65,72,86,91,96,114,116].

Instead, Sirius Red staining can be performed to evaluate collagen secretion of scaffold-seeded cells [57,76,93]. In this case, the analysis can also be followed by a quantitative measure, such as dissolving the staining and determining the solution’s absorbance [57,76]. 

Chemokines, growth factors, and pro- and anti-inflammatory cytokines released in culture medium can be also quantified by means of commercial assays and fluorescence flow fluorimetry [104]. A downregulation of key stem cell markers can be confirmed during the differentiation process [91].

Interestingly, a number of papers describe the use of an in vitro bioactivity immersion assay in a simulated body fluid solution (SBF) to evaluate the possible formation of a hydroxyapatite layer on the surface of a given biomaterial [117]. Given that hydroxyapatite is like the mineral phase of bone tissue (bone-like apatite) that is key for enabling the attachment, growth, and proliferation of precursor cells on scaffolds, this test provides an easy-to-use and low cost tool to qualitatively estimate the bone-bonding and potential mineralization abilities of a scaffold [117]. The superficial film developed on the biomaterial’s surface is usually evaluated with SEM [62,77,79,83,89,91,95,97,103,118,119,120,121,122]. This method can also be used to assess the biomaterial biodegradation: the scaffold is immersed in a SBF solution and the scaffold weight loss can be measured [91,118].

### 6.3. Chondrogenesis

Endochondral ossification, that is the process of bone formation occurring in long bone development, involves the formation of a temporary cartilage scaffold, which is gradually replaced by bone afterwards. Therefore, the effect of bone graft substitutes on chondrogenesis is also being evaluated in selected applications. 

Alcian blue staining is commonly used to label differentiated chondrocytes by determining the extent of sulfated proteoglycans [123,124,125,126]. Alternatively, toluidine blue staining [127] or safranin-O staining [128] is also used to selectively stain cartilage matrix components such as proteoglycans and glycosaminoglycans. The levels of transcripts/proteins expressed in chondrocytes [namely collagen type II alpha 1 chain (COL2A1), aggrecan (ACAN), SRY-box transcription factor 9 (SOX9), and hyaluronidase 1 (HYAL1)] can be evaluated [72,123,124,125,127,128]. 

## 7. Biomaterial Resorbability

The physiological bone remodeling process also requires the participation of osteoclasts, key players in bone resorption. Bone architecture is maintained by a combined activity between the effects of osteoblasts that form bone and those of osteoclasts that instead represent the resorbing cells in bone tissue. Several papers also aim to evaluate in vitro the important prerequisite of resorbability of bone graft substitutes, defined as the capability of biomaterials to disappear from the site of implantation over time [129]. Standard protocols are usually based on the cultivation of primary monocytes/macrophages isolated from blood samples [130,131,132] or from bone marrow [106,113,133], or of the RAW 264.7 murine macrophage cell line [133,134,135] on the scaffolds of interest, followed by the analysis of monocyte adhesion, proliferation, and osteoclast formation. The osteoclastogenesis process can be studied using different protocols.

The presence of tartrate resistant acidic phosphate (TRAP)-positive cells, representing mature osteoclasts, is usually evaluated [106,113,131,132,133,134,135]. The measure of the enzymatic activity of TRAP5b and cathepsin K (CTSK) in the medium supernatant of scaffold-seeded cells, as well as of their expression levels, are also reported [92,130,133]. In the supernatants of cells, the concentration of Mg^2+^, Ca^2+^, and PO4^3-^ ions can also be evaluated to measure chemical erosion and cell-mediated resorption [131].

The expression levels of key markers involved in osteoclast differentiation such as TRAP, TNF receptor superfamily member 11a (TNFRSF11A/RANK), TNF superfamily member 11 (TNFSF11/RANKL), nuclear factor of activated T cells 1 (NFATC1), matrix metallopeptidase 9 (MMP9), osteoclast stimulatory transmembrane protein (OCSTAMP), and dendrocyte expressed seven transmembrane protein (DCSTAMP) are also analyzed at both transcript and protein level [72,92,106,133,134,135].

The attached osteoclasts can also be removed by the analyzed biomaterial and the entity of the resorption areas can be measured [106,113,132,133]. Other parameters that can be also assessed are: (i) the activity of the intracellular carbonic anhydrase II (CA II), an early marker for osteoclast differentiation and resorption activity, [131], and (ii) the effect on the apoptosis of osteoclasts induced by the biomaterials [106].

## 8. Other Biomaterial Bioactivities

We have so far focused our attention on analyzing the effect of different supports on osteogenesis and, therefore, on precursor cells, osteoblasts, and osteoclasts. However, it is also necessary to consider and evaluate the effects that the biomaterial may have on all the other cells that populate the bone stem niche to optimize biomaterial integration as much as possible for regenerative medicine applications.

### 8.1. Inflammatory Response

Immune cells actively participate in bone homeostasis, remodeling, and regeneration. They exert an osteoinductive effect on precursor cells secreting cytokines and growth factors. Therefore, the impacts that bone-mimetic biomaterials can have on immune cells need to be considered too. Osteoimmunology is an emerging research field in tissue regeneration that deals with the bidirectional cross-talk between bone cells and the immune system that regulates the bone healing process [136]. The implantation of bone graft substitutes generally creates an inflammatory environment that is crucial to determine whether there is a successful tissue regeneration or not. Macrophages represent one of the key players in this process, and the success of biomaterial implantation can depend on the M1/M2 polarization states of macrophages.

In this regard, several studies have investigated the interaction between immune cells and biomaterials/osteogenic cells. The RAW 264.7 murine macrophage cell line [14,59,75,81,86,88,102,108,137,138,139], macrophages derived from the human monocyte THP-1 line [76], or primary bone marrow-derived monocytes [74,140], are alternatively exploited as suitable cellular models to study biomaterial-induced inflammatory responses. In addition, specific protocols are used to test immune cell adhesion, viability, and growth.

The expressions of macrophage-phenotype surface markers, as CD163, CD86, CD11c, CD206, and C-C motif chemokine receptor 7 (CCR7), of proinflammatory cytokines as tumor necrosis factor (TNF/TNFα), nitric oxide synthase 2 (NOS2/iNOS), IL1β, IL6, IL18, and C-C motif chemokine ligand 2 (CCL2), of anti-inflammatory cytokines as IL4, IL10, IL13, arginase 1 (ARG1) and transforming growth factor beta 1 (TGFB1), of growth factor genes able to promote osteogenesis, as *bone morphogenetic protein 2* (*BMP2*), *bone morphogenetic protein 6* (*BMP6*), *vascular endothelial growth factor A* (*VEGF*) and *oncostatin M* (*OSM*) in RAW 264.7, can be evaluated by flow cytometry and/or RT-qPCR and/or immunofluorescence staining and/or Western blot [14,59,74,75,76,81,86,88,101,102,108,137,138,139]. It is possible to assess the expression of inflammation-associated genes, such as *interleukin 6* (*IL6*), on osteogenic cells cultured on the scaffolds [89]. 

Moreover, the contents of TNF, TGFB1, IL6, IL10, and IL4 in the culture supernatants of macrophages can be assessed with ELISA [14,59,75,76,88,108]. 

Otherwise, the osteogenic differentiation of precursor cells and osteoblasts can be evaluated in co-culture systems, in the presence of macrophages seeded on the biomaterial of interest or using macrophage-conditioned media [59,74,75,108,137,139].

The effects of selected biomaterials on T cell proliferation and differentiation, and the subsequent impact on precursor bone cell differentiation, can also be investigated. The viability of T lymphocytes cultured in presence of the biomaterial can be assessed with standard methods (e.g., the CCK-8 assay) or by evaluating apoptosis through functional assays based on flow cytometry [141]. The differentiation process is usually evaluated by analyzing the T cell CD4^+^/CD8^+^ ratio [141]. 

### 8.2. Angiogenesis

The vascularization of the implanted bone substitute is indispensable for appropriate bone healing since vessels provide the main source of nutrients for the entire bone niche. The human umbilical vein endothelial (HUVECs) cell line is commonly used to assess the effects of bone biomaterial on vessel formation in vitro [75,105,139,140,142,143,144,145]. As an alternative, the use of human aortic endothelial cells (HAECs) is also reported [146]. The tube formation assay is a classic angiogenesis assay that can be used to assess the angiogenic differentiation of HUVECs in contact with a specific biomaterial; the tube structures as well as the total branch length can be analyzed with a contrast inverted microscope [86,105,140,142,143]. 

The expression of angiogenic related genes, such as *platelet and endothelial cell adhesion molecule 1 (PECAM1/CD31), vascular endothelial growth factor A (VEGFA), fibroblast growth factor 2 (FGF2), kinase insert domain receptor/vascular endothelial growth factor receptor 2 (KDR), C-X-C motif chemokine ligand 12 (CXCL12/SDF1A), nitric oxide synthase 3 (NOS3*), and *hypoxia inducible factor 1 subunit alpha (HIF1A)* can be analyzed by RT-qPCR and/or immunofluorescence and/or Western blot analysis and/or ELISA [75,86,105,107,139,140,142,143,144,145]. 

A migration test of endothelial cells by scratch wound healing assay on the biomaterial can be added to the set of tests aimed at assessing the angiogenic properties of a bone-substitute material [86,105,139,142,143,145].

Moreover, in this case, the reciprocal effects of precursor bone cells as well as of immune cells on angiogenesis and vice versa in the presence of the tested biomaterials can be determined by culturing different cell types simultaneously [75,105,142].

### 8.3. Antimicrobial Properties

In order for a synthetic scaffold to grant functionally effective bone regeneration and healing, the capability to impede bacterial adhesion and growth is a desirable property. Contamination of the implant represents one of the most adverse events that can compromise the success of the surgery. Some biomaterials retain inherent antimicrobial properties, while others need to be combined with other biologically active materials or molecules to enhance the requirement for antimicrobial function. Optimized protocols are used to evaluate if selected scaffolds can inhibit the adhesion and proliferation of bacteria. 

Bacteria dissociated from the biomaterial after a period of incubation can be counted and analyzed with a simple colony-forming unit (CFU) test [56,57,58,85,95,96,102,119,120,147,148,149,150,151]. 

A bacterial live/dead stain is also often performed to assess the antimicrobial potential of the scaffold [96,150]. 

A typical assay relies on analyzing the diameter of the antibacterial ring or inhibition zone produced by placing the scaffolds in contact for several hours with the surface of the LB agar plates where the bacteria are growing [57,80,97,119,121,152,153].

PCR analysis can be carried out to detect the expression of bacterial pathogenic genes [85,96]. 

Moreover, a SEM can be used to observe the presence and morphology of bacteria attached to the biomaterial [55,85,96,119,148,149,151].

Intracellular ROS production is also seldom investigated as a marker of the cell redox equilibrium that can be influenced by the biomaterial [96,150]. 

To test cell metabolism, the ATP levels of bacteria can also be evaluated using commercial kits [55].

Finally, the membrane permeability of bacteria cultured on different substrates can also be assessed using an O-nitrophenyl-β-D-galactopyranoside (ONPG) assay that allows us to measure β-galactosidase enzyme activity [55].

## 9. Animal Models for In Vivo Biomaterial Testing

Further in vivo validation is required for the complete assessment of the biological properties of engineered biomaterials for bone regeneration. Different animal models are usually exploited for this aim, with selection based upon the primary outcomes to be assessed (e.g., resorption, stability, biomechanics, etc.) and on the skeletal district (appendicular or axial skeleton, weight-bearing versus defect filling) and/or on the specific disease for which the tissue engineering strategy is designed. In this final section, we briefly summarize the animal species used in the field. A complete review of the topic is provided elsewhere and falls beyond the specific aims of this study. See [154,155] for a dedicated systematic description.

Briefly, murine models are primarily used to evaluate the long-term integration of the scaffolds [78,123,146]. Rats [55,59,62,74,75,86,96,101,106,139] and rabbits [58,125,147,152,156] find wide use for bone regenerative applications to test osteogenesis, angiogenesis, immune response, resorption, and infections after biomaterial implantation. Caprine and ovine models are larger animal models and preferred for the evaluation of large osteochondral defect repair [126,157,158,159,160]. Different procedures have been optimized to create osteochondral defects, including cranial defects, in these animal models and thus test the regenerative capabilities of selected scaffolds/composites.

Several methods can be used to analyze the proportion and characteristics of the newly formed bone tissue, including microcomputed tomography scanning [58,59,62,74,86,106,123,125,139,147] and/or immunohistological, and immunofluorescence staining [62,74,75,106,125,139,147].

## 10. Conclusions

Bone tissue engineering represents an established though still rapidly growing branch of regenerative medicine, and the literature on clinical and basic research studies in the field is extremely wide and heterogeneous. This review attempted to provide a comprehensive overview of the various tests that must be performed in order to assess a biomaterial’s suitability for bone regenerative applications. In the absence of univocal guidelines, this primer can provide both clinical and basic science researchers with a concise handbook of instructions to fulfill all needed requirements prior to introducing a novel bone substitute into the scientific landscape.

## Figures and Tables

**Figure 1:**
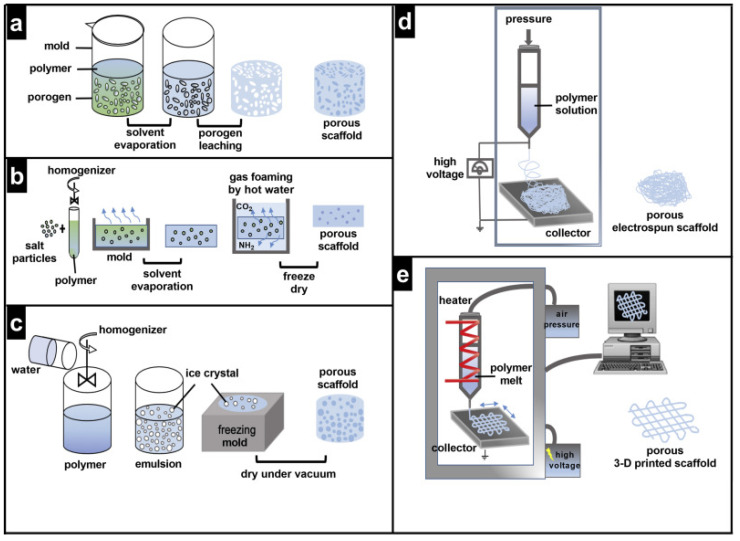
Methods for pore fabrication (**a**) Porogen leaching, (**b**) Gas foaming, (**c**) Freeze-drying, (**d**) Solution electrospinning, (**e**) Melt electrowriting and 3D printing, reproduced from [29] Creative Commons CC-BY license.

**Table 1:** Advantages and disadvantages of bone graft materials, modified with permission from [10].

Bone Graft	Advantages	Disadvantages
**Autologous**	high osteoconductivity highest degree of biological safety no risk of immune reaction	need of additional surgery
**Xenografts**	architecture and geometric structure resemble bone well documented predictable clinical outcome slow bio-absorbability preserves augmented bone volume	possible disease transmission and potential unwanted immune reactions lacks viable cells and biological components resorption rate is highly variable reduced future availability due European regulatory changes?
**Natural biomaterials**	Similarity to native extracellular matrix	Mechanical properties poor -biodegradability less controllable
**Synthetic polymers**	tuneable physicochemical properties tuneable degradability	low cell attachment timing of absorption (alteration of mechanical properties) release of acidic degradation products
**Synthetic bioceramics**	high biocompatibility osteoinductive properties chemical similarity with bone stimulation of osteoblast growth	high brittleness low ductility not predictable absorption
**Composite xenohybrid substitutes**	high similarity with human cancellous bone higher bioactivity tailored degradation rates incorporation of active biomolecules	cleaning and sterilization process partially alters biological performances limited clinical data

**Table 2:** Bone mechanical properties using uniaxial tensile stress. Reproduced under the Creative Commons CC-BY license [31].

	Name of the Mechanical Property Parameter	Bones with Osteoporosis	Bones without Osteoporosis
1	Range of the elastic region (in strain) (m/m)	0–0.0063	0–0.0043
2	Range of the plastic region (in strain) (m/m)	0.0063–0.0089	0.0043–0.0129
3	Proportional limit (in stress) (MPa)	77.0934	80.3718
4	Elastic limit (in stress) (MPa)	88.3528	98.6828
5	Failure strength (in stress) (MPa)	94.9280	116.9657
6	Brittleness coefficient (Dimensionless)	0.7079	0.3333
7	Modulus of resilience (MJ/m^3^)	0.3394	0.2450
8	Modulus of toughness (MJ/m^3^)	0.5778	1.1751
9	Modulus of elasticity (MPa)	18283.2314	27544.2425
10	Tangent modulus (MPa)	2490.2230	2118.0671
11	Strain hardening parameter (MPa)	2882.8784	2294.5076

**Table 3:** The table reports the list of genes whose expression is usually analyzed to assess the osteogenic potential of scaffold-seeded cells. The main function in osteogenesis is described for each gene (source: https://www.genecards.org/ and https://www.ncbi.nlm.nih.gov/ (accessed on 27 June 2022)).

Gene Name	Gene Symbol	Function
*RUNX family transcription factor 2*	*RUNX2*	Member of the RUNX family of transcription factors characterized by a Runt DNA-binding domain. It is fundamental for osteogenesis and skeletal morphogenesis. It acts as a scaffold for other regulatory factors involved in osteoblast maturation. Its expression increases forthwith, starting from the first steps of osteogenesis.
*bone gamma-carboxyglutamate protein (osteocalcin)*	*BGLAP*	Bone protein is extensively secreted by osteoblasts that regulates bone remodeling and energy metabolism by binding to calcium and hydroxyapatite rich in the mineral matrix.
*secreted protein acidic and cysteine rich (osteonectin)*	*SPARC*	Cysteine-rich acidic matrix-associated protein is involved in extracellular matrix synthesis and cell shape changes.
*secreted phosphoprotein 1 (osteopontin)*	*SPP1*	Secreted bone protein that binds to hydroxyapatite with high affinity, thus representing an integral part of the mineralized matrix. It is probably important for cell-matrix interaction that is involved in the attachment of osteoclasts to the mineralized bone matrix.It also plays a key role in the activation of type I immunity, acting as a cytokine, enhancing the production of interferon-gamma and interleukin-12 and reducing the production of interleukin-10.
*integrin binding sialoprotein*	*IBSP*	One of the major structural proteins of the bone matrix, synthesized by skeletal-associated cell types, including hypertrophic chondrocytes, osteoblasts, osteocytes, and osteoclasts. It constitutes approximately 12% of the non-collagenous proteins in human bone. It binds to calcium and hydroxyapatite and mediates cell attachment.
*alkaline phosphatase, biomineralization associated*	*ALPL*	A membrane bound glycosylated enzyme that is a member of the alkaline phosphatase family of proteins. It plays an essential role in bone mineralization by acting at different levels of osteogenesis.
*Sp7 transcription factor*	*SP7 (OSX)*	Bone specific transcription factor required for osteoblast differentiation and bone formation.
*bone morphogenetic protein 2*	*BMP2*	Secreted ligand of the TGF-beta (transforming growth factor-beta) superfamily of proteins. The downstream activated signal cascade leads to the recruitment and activation of SMAD family transcription factors that regulate gene expression for bone and cartilage development.
*bone morphogenetic protein 4*	*BMP4*	Another secreted ligand of the TGF-beta superfamily of proteins that activates the SMAD pathway. This protein regulates heart development and adipogenesis.
*bone morphogenetic protein 6*	*BMP6*	Like BMP2 and BMP4, this secreted protein activates SMAD signaling and regulates a wide range of biological processes, including fat and bone development.
*bone morphogenetic protein 7*	*BMP7*	Secreted ligand of the TGF-beta superfamily, which plays a role in bone, kidney, and brown adipose tissue development. This protein is also involved in ectopic bone formation and may promote fracture healing in human patients.
*collagen type I alpha 1 chain*	*COL1A1*	Pro-alpha1 chains of type I collagen are present in most connective tissues and particularly abundant in bone.
*SMAD family member 1*	*SMAD1*	Proteins belonging to the SMAD family mediate multiple signaling pathways. Specifically, SMAD1 mediates the signals of BMPs, and the activated phosphorylated form of this protein forms a complex with SMAD4, which is important for its function in transcription regulation.
*SMAD family member 3*	*SMAD3*	One of the principal master regulators of the osteogenic lineage during mesenchymal stem cell commitment. This protein forms a complex with other SMAD proteins and binds DNA, functioning as a transcription factor. For example, SMAD3 has been shown to bind to the SSP1 promoter as a sequence-specific activator.
*SMAD family member 5*	*SMAD5*	Protein activated by bone morphogenetic proteins type 1 receptor kinase and is involved in the transforming growth factor beta signaling pathway.
*SMAD family member 9*	*SMAD9*	Protein activated by bone morphogenetic proteins that interact with SMAD4.
*Transforming growth factor beta 3*	*TGFB3*	Secreted ligand of the TGF-beta superfamily of proteins that can form heterodimers with other TGF-beta family members. It is involved in embryogenesis and cell differentiation and may play a role in wound healing.

## Data Availability

Not applicable.
